# Pseudohyperkalemia: To Treat or not to Treat

**DOI:** 10.7759/cureus.3570

**Published:** 2018-11-10

**Authors:** Akriti G Jain, Abubakar Tauseef, Syed A Hasan, Sanjay K Jain, Mohammed Wazir, Umair Majeed

**Affiliations:** 1 Internal Medicine, Florida Hospital, Orlando, USA; 2 Internal Medicine, Dow University of Health Sciences, Karachi, PAK; 3 Family Medicine, Florida Hospital, Orlando, USA

**Keywords:** cll-chronic lymphocytic leukemia, hyperkalemia

## Abstract

Chronic lymphocytic leukemia (CLL) is characterized by excessive production of abnormal lymphocytes in the bone marrow. It usually presents as hepatosplenomegaly and lymphadenopathy along with constitutional symptoms of fever, tiredness, and weight loss. Pseudohyperkalemia may occur in patients with extreme leukocytosis. High serum and plasma potassium levels have been seen in pseudohyperkalemia. Whole blood potassium determination will usually help lead to a correct diagnosis. It is important to diagnose this condition early so that the patients are not inappropriately treated. We aim to highlight that hyperkalemia in CLL patients should only be treated if the patient is symptomatic or if the patient shows cardiac effects of hyperkalemia on electrocardiogram.

## Introduction

Pseudohyperkalemia is a rise in serum potassium with concurrently normal plasma potassium concentration. It is known to be an in-vitro phenomenon that was first described 50 years ago [1]. It has been shown that the use of vacuum tubes for blood draws, pneumatic tube for transport or even simply shaking the syringe into which blood is drawn can lead to lysis of the fragile malignant white cells and lead to spuriously high measurement of potassium [2]. We present a case of chronic lymphocytic leukemia (CLL) with pseudohyperkalemia.

## Case presentation

**Figure 1:**
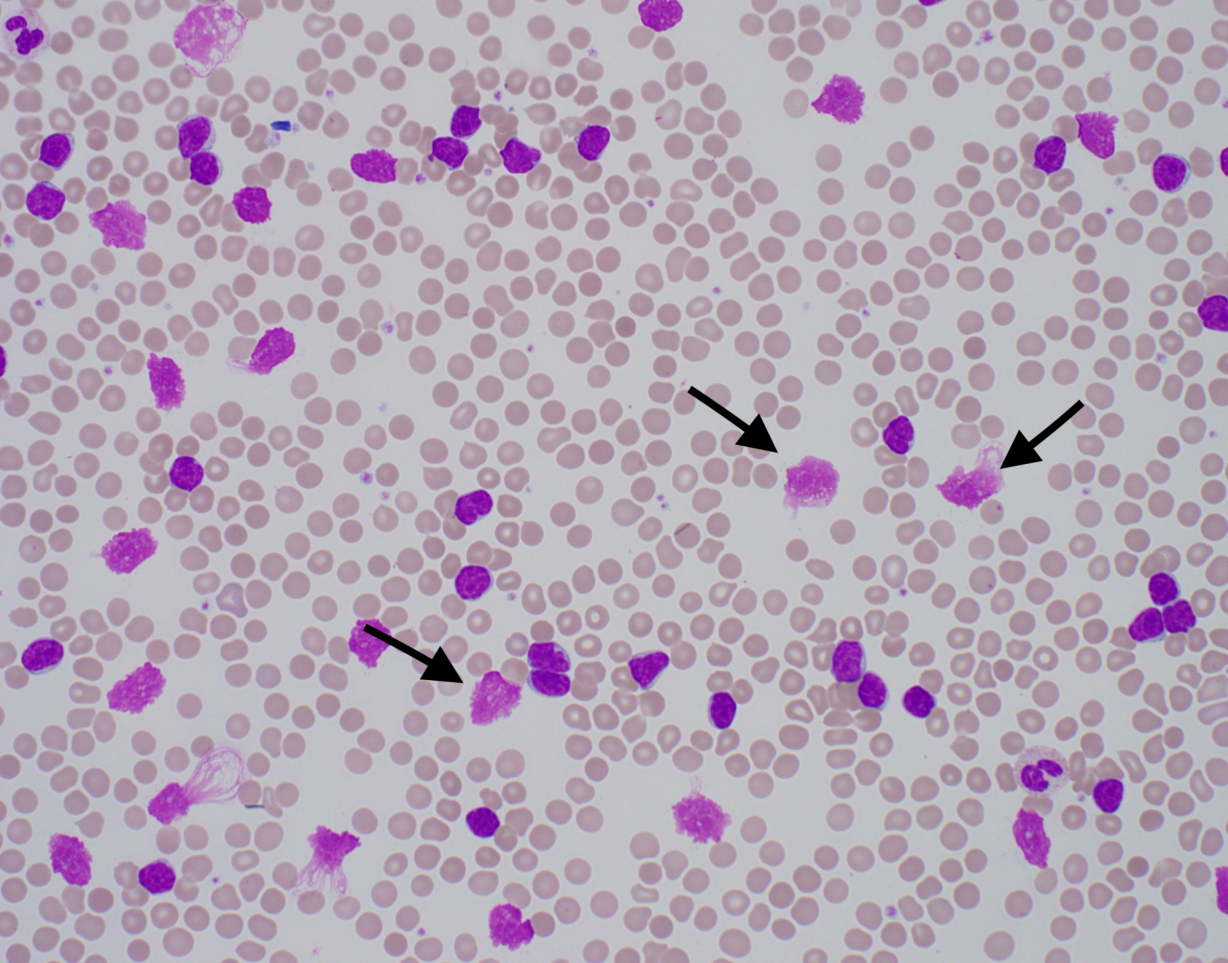
Peripheral blood film showing smudge cells.

A 57-year-old male presented with left upper extremity swelling and pain for five days. He had been recently discharged from another facility after treatment of left lower extremity cellulitis. On that admission he was told that his white blood cell (WBC) count was more than 200 X 10^3^ and that they were suspecting CLL, but before further testing could be done to confirm the diagnosis he left against medical advice. On further questioning, the patient was found to be homeless, with no insurance or money, and he said that he lived on a road crossing opposite the hospital. He did not have any significant past medical or surgical history. On examination he was afebrile and did not have tachycardia. His left upper extremity was swollen from below the elbow to the hand, was red, indurated, and tender. His left lower extremity was also swollen and indurated but did not show any redness or tenderness. Initial labs revealed a WBC of 256 with 87.5% atypical lymphocytes, hemoglobin of 13.4 g/dl, a platelet count of 166 x 10^9^/L. His electrolytes were significant for a potassium level of 5.4 meq/L. A peripheral blood smear showed smudge cells (Figure 1). Further testing showed enlarged lymph nodes within retroperitoneal, mesenteric, pelvic and inguinal distribution, as well as axillary and right hilar areas. Flow cytometry revealed CLL with monoclonal B cells (CD19+, CD20DIM+, CD5+, CD23+, CD10-, FMC7-, CD38-, ZAP70- SURFACE KAPPA DIM+). Over the course of the hospital stay the labs of the patient showed a potassium level as high as 8 meq/L. At other times it ranged between 6 meq/L and 8 meq/L, while the patient remained asymptomatic and the electrocardiogram did not show any changes. A decision was made to not treat the patient's potassium since it was concluded to be falsely elevated. The patient was discharged from the hospital on cephalexin for cellulitis with outpatient follow-up with hematology-oncology.

## Discussion

The reason for pseudohyperkalemia accompanying exceptionally elevated leukocyte count is most likely malignant cell lysis with the release of intracellular potassium. The cause of this cell destruction can be multifaceted. At first the cell lysis was thought to be secondary to clotting, but current reports suggest mechanical problems may also play a significant role. The utilization of vacuum tubes [3], pneumatic tube transport [4], tourniquet usage [5], and extended incubation have all been implicated in leading to cell lysis and pseudohyperkalemia. Re-centrifugation after storage in gel separator tubes has also been reported to cause pseudohyperkalemia, and the degree of hyperkalemia was noted to increase as storage times increased [6]. The heightened fragility of leukemic white cells is considered to contribute to their tendency to lyse and liberate potassium. Depletion of metabolites may contribute to the hyperkalemia associated with prolonged storage [7].

The diagnosis of pseudohyperkalemia can be made by comparing the level of serum and plasma potassium with an expectation that the serum potassium will be higher than the plasma potassium [8]. In this method, a sample of plasma is put in a tube that contains heparin as the anticoagulant (although it can also be collected with other additives, such as ethylenediamine tetra-acetic acid (EDTA) and citrate), whereas serum is collected in a tube that does not have heparin or other anticoagulants. In the clotting process, platelets undergo aggregation and degranulation while also releasing potassium [9]. As a result, serum potassium is higher compared to plasma potassium levels [10]. Moustafa et al. [11] conducted a study in which they described the causes of pseudohyperkalemia in CLL patients as (a) negative pressure in a vacutainer causing destruction of the fragile leukemic blasts (b) the large number of leukocytes might amplify the leakage effect of potassium due to coagulation from cells in non-heparinized specimens (c) centrifugation of serum samples can lead to extensive blasts destruction as opposed to whole blood potassium samples. Abraham et al. [12] also reported a leukemic patient with what they called “reverse pseudohyperkalemia,” that is, pseudohyperkalemia in plasma but not serum or whole blood. Furthermore, Lee et al. [13] reported four CLL patients in whom hyperkalemia was noted in serum, but not in plasma or on analysis of whole blood utilizing a blood gas analyzer. In a study conducted at MD Anderson Cancer Center, 37 paired samples were taken which showed that serum potassium values were significantly greater than those obtained from plasma on average [14].

Our patient's presentation and course highlights the dilemma involved in the diagnosis and treatment of pseudohyperkalemia in a patient with a greatly elevated WBC. Despite a high potassium ranging from 5 meq/L-8 meq/L, our patient did not show any clinical signs of hyperkalemia. Multiple EKGs did not show electrocardiographic signs of high potassium. He had a remarkably raised WBC, and the blood sample was sent to the lab via the pneumatic tube system. Our patient could have received multiple treatments for high potassium including insulin, beta-agonists, and kayexalate. Inappropriate treatment, especially in excess can potentially lead to detrimental effects including hypokalemia and arrhythmogenic effects on the heart. Hence it is imperative to recognize when potassium is spuriously elevated and hence might not require any measures to bring it lower.

In patients with a diagnosed case of CLL, the presence of leukocytosis is common, so the clinician should be aware of high chances of spurious hyperkalemia. The recommendation in this scenario is to immediately obtain a potassium level by blood gas analysis, as this is a quick and reliable test. It has been proposed that there should be a mechanism through which the hospital information system flags elevated potassium results in patients with leukocyte count above 100 x 103/mL bearing in mind the relationship between remarkable leukocytosis and pseudohyperkalemia [13] and hence lead to less nursing and medical errors.

## Conclusions

Spurious elevation of blood potassium levels is known to occur in cases of extreme leukocytosis and should not be treated unless the patient is symptomatic or has EKG changes. The treatment of hyperkalemia multiple times in such cases can lead to deleterious effects. It has been suggested to confirm the potassium levels by obtaining an arterial sample if in doubt, especially in cases of WBC count > 100 X 10^3^. Our case re-emphasizes the importance of treating the patient as a whole and not as mere laboratory values.
